# Isatuximab plus carfilzomib and dexamethasone in patients with relapsed multiple myeloma based on prior lines of treatment and refractory status: IKEMA subgroup analysis

**DOI:** 10.1002/ajh.26602

**Published:** 2022-06-04

**Authors:** Meletios A. Dimopoulos, Philippe Moreau, Bradley Augustson, Nelson Castro, Tomas Pika, Sosana Delimpasi, Javier De la Rubia, Angelo Maiolino, Tony Reiman, Joaquin Martinez‐Lopez, Thomas Martin, Joseph Mikhael, Kwee Yong, Marie‐Laure Risse, Gaelle Asset, Sylvia Marion, Roman Hajek

**Affiliations:** ^1^ The National and Kapodistrian University of Athens Athens Greece; ^2^ Department of Hematology University Hospital Hôtel‐Dieu Nantes France; ^3^ Sir Charles Gairdner Hospital Perth Western Australia Australia; ^4^ Hospital de Cancer de Barretos São Paulo Brazil; ^5^ Department of Hemato‐Oncology University Hospital Olomouc Olomouc Czech Republic; ^6^ Department of Haematology General Hospital of Athens Athens Greece; ^7^ Hematology Department University Hospital La Fe Valencia Spain; ^8^ Instituto COI de Ensino e Pesquisa and Faculdade de Medicina Universidade Federal do Rio de Janeiro Rio de Janeiro Brazil; ^9^ Department of Oncology, Saint John Regional Hospital Dalhousie University and University of New Brunswick Saint John New Brunswick Canada; ^10^ Departamento de Hematología Hospital 12 de Octubre, Complutense University Madrid Spain; ^11^ UCSF Helen Diller Family Comprehensive Cancer Center San Francisco California USA; ^12^ Translational Genomics Research Institute, City of Hope Cancer Center Phoenix Arizona USA; ^13^ Department of Haematology University College Hospital London UK; ^14^ Sanofi Vitry‐sur‐Seine France; ^15^ Sanofi Chilly‐Mazarin France; ^16^ Sanofi Cambridge Massachusetts USA; ^17^ Department of Hemato‐Oncology University Hospital Ostrava Ostrava Czech Republic; ^18^ Department of Hemato‐Oncology, Faculty of Medicine University of Ostrava Ostrava Czech Republic

To the Editor:

Patients with multiple myeloma (MM) often relapse or become refractory to successive lines of therapy (LOT), warranting more effective treatments. Novel treatments have improved outcomes; however, MM is associated with a significant patient burden. Patients who are refractory to immunomodulatory drugs and proteasome inhibitors (PIs) have poor prognosis. Many patients with MM are exposed to lenalidomide or bortezomib in early LOT; those refractory to these agents are challenging to treat and represent a high unmet medical need.^1^

Based on the Phase 3 ICARIA‐MM study (NCT02990338),^2^ isatuximab (Sarclisa), a CD38 monoclonal antibody, is approved in combination with pomalidomide and dexamethasone (Isa‐Pd) for adult patients with relapsed and refractory MM (RRMM) who have received ≥2 prior therapies, including lenalidomide and a PI. Based on the IKEMA study (NCT03275285),^3^ to date, isatuximab in combination with carfilzomib and dexamethasone (Isa‐Kd) is approved in the United States for adult patients with relapsed or refractory MM with 1–3 prior LOT, in the European Union for adult patients with MM with ≥1 prior therapy, and in Japan for adult patients with relapsed or refractory MM with one prior treatment.

IKEMA demonstrated that, in patients with relapsed MM, Isa‐Kd significantly improved progression‐free survival (PFS) compared with Kd (hazard ratio [HR] 0.53; 99% confidence interval [CI] 0.32–0.89; *p* = .0007), with a clinically meaningful increase in minimal residual disease (MRD) negativity and complete response (CR) rates in the intent‐to‐treat population, and a manageable safety profile.^3^ We conducted a prespecified subgroup analysis of IKEMA to evaluate the efficacy and safety of Isa‐Kd versus Kd according to number of prior LOT (1 vs. >1), and an exploratory subgroup analysis based on refractoriness to two frequently used front‐line agents, lenalidomide and bortezomib.

Randomized patients (*N* = 302) received Isa‐Kd (*n* = 179) or Kd (*n* = 123). Subgroup analyses were conducted by number of prior LOT (1 vs. >1) as entered by the investigator at randomization and by refractory status (defined as: (i) reason for discontinuation was progression, or (ii) progression ≤60 days posttreatment, or (iii) best response was stable disease or progressive disease). The study design and procedures are described in Supporting Information.

In the overall population, patients received a median (range) of 2 (1–4) prior LOT in both treatment arms; 44.4% of patients received 1 prior line, 32.8% were lenalidomide‐refractory, and 30.1% were bortezomib‐refractory. Table S1 shows patient baseline characteristics in each subgroup. Compared with Kd, more patients with Isa‐Kd were aged ≥75 years in the 1 prior line subgroup, fewer patients were International Staging System Stage I in the >1 prior line subgroup, and more were aged <65 years in the lenalidomide‐refractory subgroup.

Exposure to study treatment was longer with Isa‐Kd than Kd. The median (range) number of treatment cycles with Isa‐Kd versus Kd was: 20.0 (1–25) versus 16.5 (1–28), 1 prior line; 18.0 (1–27) versus 12.5 (1–26), >1 prior line; 14.0 (1–27) versus 11.5 (1–28), lenalidomide‐refractory; 13.5 (1–26) versus 13.0 (1–28), bortezomib‐refractory. More patients with Isa‐Kd than Kd received ≥18 cycles in all subgroups: 65.8% versus 48.1%, 1 prior line; 51.0% versus 32.4%, >1 prior line; 43.9% versus 33.3%, lenalidomide‐refractory; 38.5% versus 25.6%, bortezomib‐refractory. These results are consistent with IKEMA overall population where a longer treatment duration was reported with Isa‐Kd (median [range] number of cycles 19.0 [1–27] and 57.6% patients with ≥18 cycles) than Kd (14.5 [1–28] and 39.3% patients with ≥18 cycles).

Consistent with IKEMA overall population, PFS improvement was observed with Isa‐Kd versus Kd across all subgroups analyzed, regardless of number of prior LOT (HR 0.59 [95% CI, 0.31–1.12], 1 prior line; HR 0.48 [95% CI, 0.29–0.78], >1 prior line) or refractory status (HR 0.60 [95% CI, 0.34–1.1], lenalidomide‐refractory; HR 0.69 [95% CI, 0.35–1.39], lenalidomide‐refractory at last regimen; HR 0.62 [95% CI, 0.33–1.16], bortezomib‐refractory; HR 0.38 [95% CI, 0.16–0.92], bortezomib‐refractory at last regimen; Figure 1A). The *p* values for interaction suggest no interaction with any of the parameters evaluated. The PFS‐event‐free probability at 18 months for Isa‐Kd versus Kd was: 77% versus 64%, 1 prior line; 68% versus 45%, >1 prior line; 53% versus 31%, lenalidomide‐refractory patients; and 63% versus 43%, bortezomib‐refractory (Table S2).

**FIGURE 1:**
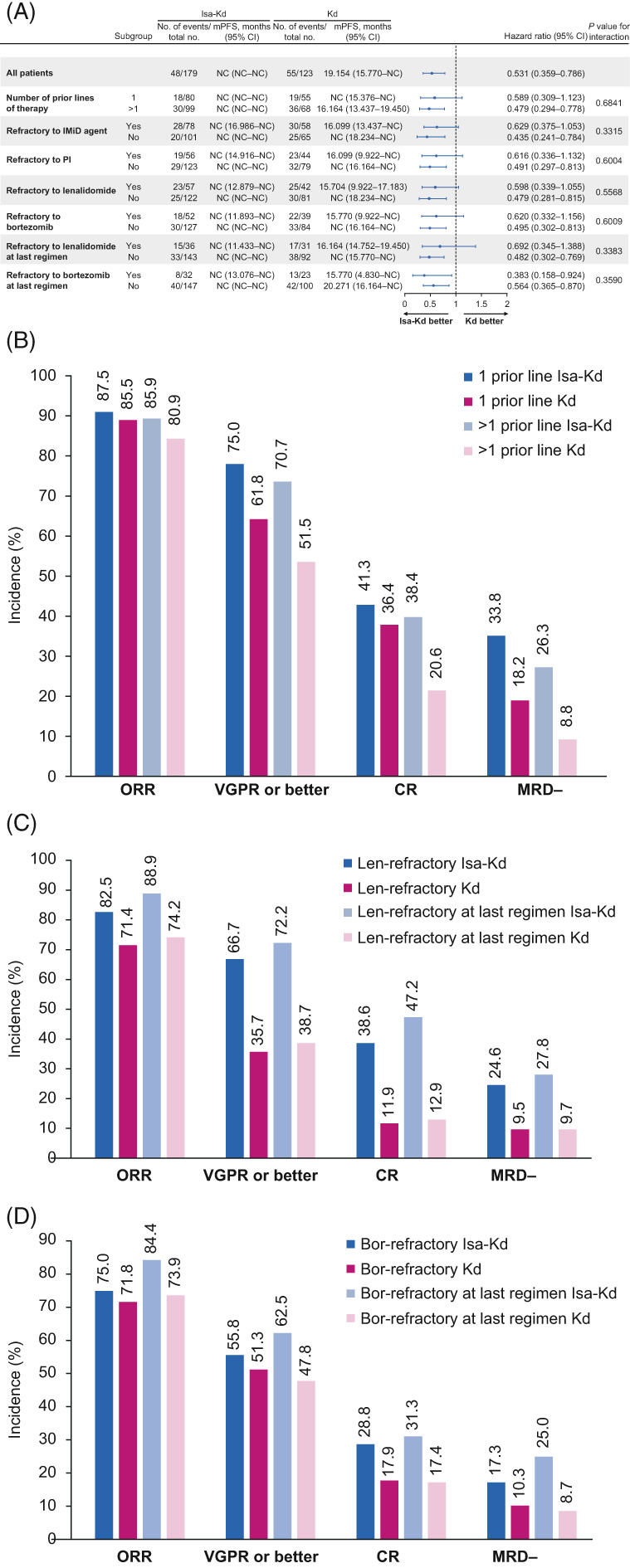
Efficacy with Isa‐Kd versus Kd. (A) PFS by number of prior lines of therapy and refractory status; depth of response by (B) number of prior lines of therapy, (C) lenalidomide‐refractory status, or (D) bortezomib‐refractory status. Bor, bortezomib; CI, confidence interval; CR, complete response; d, dexamethasone; IMiD, immunomodulatory drug; Isa, isatuximab; K, carfilzomib; Len, lenalidomide; mPFS, median progression‐free survival; MRD−, minimal residual disease negativity; NC, not calculated; ORR, overall response rate; PFS, progression‐free survival; PI, proteasome inhibitor; VGPR, very good partial response

Overall response rate (ORR) in IKEMA overall population was high in both treatment groups with no statistically significant difference (87%, Isa‐Kd vs. 83%, Kd; one‐sided *p* = 0.19); thus, *p* values of subsequent key secondary endpoints (≥very good partial response [VGPR] and MRD negativity rates) were provided for descriptive purposes only.^3^ Similar results were observed irrespective of number of prior LOT (Figure 1B; 87.5% vs. 85.5%, 1 prior line; 85.9% vs. 80.9%, >1 prior line), but a trend toward higher ORR with Isa‐Kd versus Kd was seen in refractory subgroups (Figure 1C,D; 82.5% vs. 71.4%, lenalidomide‐refractory; 88.9% vs. 74.2%, lenalidomide‐refractory at last regimen; 75.0% vs. 71.8%, bortezomib‐refractory; 84.4% vs. 73.9%, bortezomib‐refractory at last regimen).

Consistent with overall population (≥VGPR 73% vs. 56%, *p* = 0.0011; MRD negativity 29.6% vs. 13.0%, *p* = 0.0004),^3^ numerically and clinically meaningful higher ≥VGPR and MRD negativity rates, respectively, with Isa‐Kd versus Kd were observed across all subgroups: 1 prior line (75.0% vs. 61.8% and 33.8% vs. 18.2%), >1 prior line (70.7% vs. 51.5% and 26.3% vs. 8.8%), lenalidomide‐refractory (66.7% vs. 35.7% and 24.6% vs. 9.5%), lenalidomide‐refractory at last regimen (72.2% vs. 38.7% and 27.8% vs. 9.7%), bortezomib‐refractory (55.8% vs. 51.3% and 17.3% vs. 10.3%), and bortezomib‐refractory at last regimen (62.5% vs. 47.8% and 25.0% vs. 8.7%; Figure 1). A clinically meaningful difference in CR rates with Isa‐Kd versus Kd was observed for >1 prior line subgroup (38.4% vs. 20.6%) and 1 prior line subgroup (41.3% vs. 36.4%; Figure 1). Similarly, a clinically meaningful difference in CR rates with Isa‐Kd versus Kd was also observed in refractory subgroups: lenalidomide‐refractory (38.6% vs. 11.9%), lenalidomide‐refractory at last regimen (47.2% vs. 12.9%), bortezomib‐refractory (28.8% vs. 17.9%), and bortezomib‐refractory at last regimen (31.3% vs. 17.4%).

The incidence of patients with all‐grade treatment‐emergent adverse events (TEAEs) in all subgroups was similar to IKEMA safety population^3^ (97.2%, Isa‐Kd vs. 95.9%, Kd; Table S3), with infusion‐related reactions being the most frequent (Tables S4 and S5). Other most common TEAEs reported more frequently (≥10% patients) with Isa‐Kd versus Kd included pneumonia and bronchitis in 1 prior line; upper respiratory infection, fatigue, and vomiting in >1 prior line (Table S4); diarrhea, cough, hypertension, fatigue, dyspnea, upper respiratory tract infection, constipation, bronchitis, arthralgia, and nausea in lenalidomide‐refractory; and cough, fatigue, and bronchitis in bortezomib‐refractory subgroups (Table S5).

The incidence of patients with Grade ≥3 TEAEs was higher with Isa‐Kd versus Kd across all subgroups (77.2% vs. 64.8%, 1 prior line; 76.5% vs. 69.1%, >1 prior line; 73.7% vs. 61.9%, lenalidomide‐refractory; 76.9% vs. 66.7%, bortezomib‐refractory), and consistent with overall safety population^3^ (76.8% vs. 67.2%; Table S3).^3^ The most frequent Grade ≥3 TEAEs were hypertension and pneumonia, with similar incidences between treatment arms in all subgroups (Tables S4 and S5).

The incidence of patients with serious TEAEs with Isa‐Kd versus Kd was similar to that in the overall population^3^ (59.3% vs. 57.4%) in all subgroups, except in 1 prior line (62.0% vs. 48.1%) and lenalidomide‐refractory (59.6% vs. 50.0%) subgroups (Table S3). Grade 5 TEAEs occurred in 3.8% versus 0% in 1 prior line, 3.1% versus 5.9% in >1 prior line, 3.5% versus 4.8% in lenalidomide‐refractory, and 1.9% versus 7.7% in bortezomib‐refractory patients.

The incidence of patients with TEAEs leading to discontinuations was lower or similar with Isa‐Kd versus Kd in all subgroups (8.9% vs. 11.1%, 1 prior line; 8.2% vs. 16.2%, >1 prior line; 7.0% vs. 11.9%, lenalidomide‐refractory; 3.8% vs. 17.9%, bortezomib‐refractory; Table S3).

The current analysis strongly supports similar treatment benefit of Isa‐Kd versus Kd on PFS and depth of response regardless of number of prior lines or lenalidomide‐ or bortezomib‐refractory status versus the control arm Kd, which has shown in ENDEAVOR subgroup analysis to be an efficient treatment in lenalidomide‐ or bortezomib‐exposed patients, irrespective of number or type of prior LOT, with improved outcomes versus bortezomib‐dexamethasone.^4^ CANDOR reported favorable benefit‐to‐risk profile of another CD38 antibody, daratumumab, plus Kd versus Kd in patients with RRMM, regardless of number of prior lines (1 vs. ≥2) or refractoriness to bortezomib/ixazomib or lenalidomide.^5^ One key difference between these studies is the lack of M‐protein interference assay for isatuximab; CR was assessed without correction for M‐protein interference and is likely underestimated in IKEMA. The clinical significance of numerical differences observed between IKEMA and CANDOR has not been elucidated. Notably, a similar ICARIA‐MM subgroup analysis showed that Isa‐Pd improved PFS and ORR regardless of number of prior LOT and in patients who were lenalidomide‐refractory, lenalidomide‐refractory at last line, and double‐refractory to lenalidomide and PIs.^2^, ^6^

Limitations of the current study are exclusion of daratumumab‐treated patients and a relatively small number of patients owing to subgroup analysis (limiting the statistical analysis power). However, the efficacy and safety benefits of Isa‐Kd in patients with relapsed MM were seen irrespective of number of prior LOT, or lenalidomide‐ or bortezomib‐refractory status and were consistent with IKEMA overall population. Isa‐Kd is a new treatment option for patients with relapsed MM, particularly in the difficult‐to‐treat lenalidomide‐ and bortezomib‐refractory patients.

## AUTHOR CONTRIBUTIONS

Marie‐Laure Risse was responsible for study oversight. Philippe Moreau and Thomas Martin were coprincipal investigators of the study. Meletios A. Dimopoulos, Bradley Augustson, Nelson Castro, Tomas Pika, Sosana Delimpasi, Javier De la Rubia, Angelo Maiolino, Tony Reiman, Joaquin Martinez‐Lopez, Joseph Mikhael, Kwee Yong, and Roman Hajek were investigators in the study and contributed to data acquisition and analysis. Philippe Moreau, Thomas Martin, Marie‐Laure Risse, and Gaelle Asset designed the study. Marie‐Laure Risse, Gaelle Asset, and Sylvia Marion contributed to analysis and interpretation of data for the work. All authors revised the work for important intellectual content and assume responsibility for data integrity and the decision to submit this manuscript for publication, had full access to the study data, edited and reviewed manuscript drafts, and approved the final version for submission.

## CONFLICT OF INTEREST

Roman Hajek: Consulting or Advisory role—Takeda, Amgen, Celgene, Abbvie, Bristol‐Myers Squibb, PharmaMar, Janssen‐Cilag, Novartis; Speakers' Bureau—Takeda, Amgen; research funding—Novartis, Bristol‐Myers Squibb, Amgen, Celgene, Takeda. Philippe Moreau: Consulting or Advisory role—Celgene, Janssen, Amgen, GlaxoSmithKline, Sanofi, Abbvie; Honoraria—Celgene, Janssen‐Cilag, Amgen, GlaxoSmithKline, Sanofi, Abbvie. Sosana Delimpasi: Consulting or Advisor role—Janssen, Takeda, Amgen; Speakers' Bureau—Janssen, Takeda, Amgen. Javier De la Rubia: Speakers' Bureau—Amgen, Bristol‐Myers Squibb; Honoraria—Amgen, Celgene, Takeda, Janssen, Sanofi, Bristol‐Myers Squibb. Tony Reiman: Research funding—Sanofi, Terry Fox Research Institute, Canadian Cancer Society, New Brunswick Health Research Foundation, New Brunswick Foundation for Innovation, Canadian Institutes of Health Research; Hematology Disease Site Co‐Chair–Canadian Cancer Trials Group. Joaquin Martinez‐Lopez: Honoraria—Sanofi, Janssen, Novartis, Bristol‐Myers Squibb, Incyte, Roche; research funding—Janssen, Novartis, Bristol‐Myers Squibb, Incyte, Roche; nonfinancial support—Janssen, Novartis, Bristol‐Myers Squibb. Thomas Martin: Consulting or advisory role—Juno Therapeutics, GlaxoSmithKline; research funding—Sanofi, Amgen, Janssen. Joseph Mikhael: Honoraria—Amgen, Karyopharm Therapeutics, Sanofi, Janssen, Celgene, GlaxoSmithKline, Takeda. Marie‐Laure Risse, Gaelle Asset, and Sylvia Marion are employed by Sanofi and may have stock and/or stock options in the company. Meletios A. Dimopoulos: Consulting or advisory role—Amgen, Janssen‐Cilag, Takeda, Bristol‐Myers Squibb, Beigene; honoraria—Amgen, Takeda, Janssen‐Cilag, Bristol‐Myers Squibb, Beigene.

## Supporting information


**Appendix S1** Supporting Information

## Data Availability

Qualified researchers can request access to patient‐level data and related study documents including the clinical study report, study protocol with any amendments, blank case report forms, statistical analysis plan, and dataset specifications. Patient‐level data will be anonymized, and study documents will be redacted to protect the privacy of trial participants. Further details on Sanofi's data‐sharing criteria, eligible studies, and process for requesting access are at: https://www.vivli.org.
