# Supplementary material for: Isatuximab plus carfilzomib and dexamethasone in patients with relapsed multiple myeloma based on prior lines of treatment and refractory status: IKEMA subgroup analysis
Source: Am J Hematol. 2022 Jun 4;98(1):E15–9. doi: 10.1002/ajh.26602 (PMC10084376; doi:10.1002/ajh.26602)
Supplement: Supplementary file 1 — Appendix S1 Supporting Information [file AJH-98-E15-s001.docx]

**Isatuximab plus carfilzomib and dexamethasone in patients with relapsed multiple myeloma based on prior lines of treatment and refractory status: IKEMA subgroup analysis**

**Supplementary Materials:**

[METHODS 2](#_Toc100047996)

[Study design and participants 2](#_Toc100047997)

[Procedures 2](#_Toc100047998)

[Outcomes 3](#_Toc100047999)

[Statistical analysis 4](#_Toc100048000)

[Table S1. Baseline patient characteristics by number of prior lines and refractory status 5](#_Toc100048001)

[Table S2. PFS probability by number of prior lines and refractory status 8](#_Toc100048002)

[Table S3. Safety summary by number of prior lines of therapy and refractory status 9](#_Toc100048003)

[Table S4. TEAEs (occurring in ≥ 20% patients) by number of prior lines of therapy (safety population) 10](#_Toc100048004)

[Table S5. TEAEs (occurring in ≥ 20% patients) by refractory status (safety population) 13](#_Toc100048005)

[References 16](#_Toc100048006)

# METHODS

## Study design and participants

The IKEMA study has previously been described by Moreau et al.^1^ In brief, this was a prospective, multinational, randomized, open-label, parallel-group study of isatuximab-carfilzomib-dexamethasone versus carfilzomib-dexamethasone in patients with relapsed multiple myeloma (MM). This study was conducted in accordance with the Declaration of Helsinki and the International Conference on Harmonization Guidelines for Good Clinical Practice. The study protocol was approved by an institutional ethics committee or independent review board at all participating study centers. All patients provided written informed consent.

The IKEMA study included adult patients with relapsed MM who had 1-3 prior lines of therapy (LOT), measurable evidence of disease (serum M-protein ≥0.5 g/dL and/or urine M-protein ≥200 mg/24 h) and had an Eastern Cooperative Oncology Group score ≤ 2. Patients who were refractory to anti-CD38 monoclonal antibody therapy or with prior carfilzomib exposure were excluded. Randomization was stratified by number of previous lines of therapy (1 vs. > 1) and Revised International Staging System (R-ISS; stage I or II vs. III vs. not classified), at study entry.

## Procedures

A total of 302 patients were randomized 3:2 to receive Isa-Kd (n = 179) or Kd (n = 123) and stratified by number of previous LOT (1 vs. >1) and R-ISS stage I or II vs. III vs. not classified at study entry. In the experimental arm (Isa-Kd), patients received Isa 10 mg/kg intravenously on days 1, 8, 15, and 22 in the first 28-day cycle, then every 2 weeks. Patients in both arms received intravenous carfilzomib (20 mg/m² on days 1 and 2 of the first cycle, then 56 mg/m^2^ on days 8, 9, 15, and 16 of the first cycle and days 1, 2, 8, 9, 15, and 16 of subsequent cycles), and intravenous or oral dexamethasone (20 mg on days 1, 2, 8, 9, 15, 16, 22, and 23 in each cycle). Treatment was continued until disease progression, unacceptable adverse events occurred or other discontinuation criteria were met.

M-protein was assessed by central laboratory. Minimal residual negativity (MRD) was assessed by next generation sequencing Adaptive clonoSEQ Assay (Adaptive Biotechnologies, Seattle, WA, USA) at 10^-5^ sensitivity by central laboratory. Complete response (CR) was assessed without correction for M-protein interference.

Safety was assessed in patients who received at least one dose of treatment. Adverse events (AEs) were recorded until 30 days after the last administration of study treatment and graded according to the National Cancer Information Center Common Terminology Criteria for AEs (NCI-CTCAE) version 4.03.

## Outcomes

The primary endpoint was progression-free survival (PFS), defined using the International Myeloma Working Group criteria for progression and disease response evaluation.^2^ PFS was assessed by a blinded independent response committee based on central laboratory M-protein quantification, local bone marrow aspiration when needed, and central radiologic review.

Key secondary endpoints included overall response rate (ORR), very good partial response or better (≥ VGPR), MRD negativity, CR rates, and overall survival.

## Statistical analysis

A prespecified interim analysis (cutoff date: February 7, 2020) was conducted on the intent-to-treat population and utilized for this patient subgroup analysis. Subgroup analysis according to the number of prior lines was also prespecified. Exploratory analyses were conducted in lenalidomide-refractory and bortezomib-refractory patients. Median PFS and 95% confidence intervals were calculated by the Kaplan-Meier method. Hazard ratio estimates for subgroup analyses were determined using a non-stratified Cox proportional hazard model with terms for the factor, treatment, and their interaction. The test for the interaction was performed at the 10% alpha level for descriptive purposes.

The safety results were summarized by actual treatment received in patients who had at least one dose of treatment (safety population). The analyses of the safety variables were descriptive.

# Table S1. Baseline patient characteristics by number of prior lines and refractory status

|  | **1 prior line** | | **>1 prior line** | | | **Len-refractory** | | | | **Bor-refractory** | | | |
| --- | --- | --- | --- | --- | --- | --- | --- | --- | --- | --- | --- | --- | --- |
|  | **Isa-Kd**  **(n = 80)** | **Kd**  **(n = 55)** | **Isa-Kd**  **(n = 99)** | **Kd**  **(n = 68)** | | **Isa-Kd**  **(n = 57)** | | **Kd**  **(n = 42)** | | **Isa-Kd**  **(n = 52)** | | **Kd**  **(n = 39)** | |
| **Age in years, median (range)** | 66  (37–86) | 63  (40–75) | 64  (38–83) | | 64  (33–90) | | 64  (38–83) | | 68  (33–80) | | 64  (38–83) | | 63  (33–90) |
| **Age group in years** | | | | | | | | | | | | | |
| <65 | 35 (43.8) | 32 (58.2) | 53 (53.5) | | 34 (50.0) | | 30 (52.6) | | 17 (40.5) | | 29 (55.8) | | 21 (53.8) |
| ≥65 to <75 | 35 (43.8) | 22 (40.0) | 39 (39.4) | | 25 (36.8) | | 22 (38.6) | | 20 (47.6) | | 18 (34.6) | | 12 (30.8) |
| ≥75 | 10 (12.5) | 1 (1.8) | 7 (7.1) | | 9 (13.2) | | 5 (8.8) | | 5 (11.9) | | 5 (9.6) | | 6 (15.4) |
| **ISS stage at study entry** | | | | | | | | | | | | | |
| Stage I | 48 (60.0) | 36 (65.5) | 41 (41.4) | | 35 (51.5) | | 28 (49.1) | | 18 (42.9) | | 19 (36.5) | | 18 (46.2) |
| Stage II | 23 (28.8) | 13 (23.6) | 40 (40.4) | | 18 (26.5) | | 21 (36.8) | | 14 (33.3) | | 19 (36.5) | | 14 (35.9) |
| Stage III | 9 (11.3) | 6 (10.9) | 17 (17.2) | | 14 (20.6) | | 7 (12.3) | | 9 (21.4) | | 14 (26.9) | | 6 (15.4) |
| Unknown | 0 | 0 | 1 (1.0) | | 1 (1.5) | | 1(1.8) | | 1 (2.4) | | 0 | | 1 (2.6) |
| **R-ISS stage at study entry^a^** | | | | | | | | | | | | | |
| I or II | 71 (88.8) | 48 (87.3) | 84 (84.8) | | 57 (83.8) | | 49 (86.0) | | 34 (81.0) | | 40 (76.9) | | 32 (82.1) |
| III | 7 (8.8) | 5 (9.1) | 11 (11.1) | | 8 (11.8) | | 6 (10.5) | | 5 (11.9) | | 9 (17.3) | | 5 (12.8) |
| Not classified | 2 (2.5) | 2 (3.6) | 4 (4.0) | | 3 (4.4) | | 2 (3.5) | | 3 (7.1) | | 3 (5.8) | | 2 (5.1) |
| **Cytogenetic risk^b^ at study entry** | | | | | | | | | | | | | |
| High-risk CA | 23 (28.8) | 12 (21.8) | 19 (19.2) | | 19 (27.9) | | 13 (22.8) | | 12 (28.6) | | 13 (25.0) | | 12 (30.8) |
| Standard-risk CA | 52 (65.0) | 35 (63.6) | 62 (62.6) | | 43 (63.2) | | 36 (63.2) | | 25 (59.5) | | 31 (59.6) | | 22 (56.4) |
| Unknown or missing | 5 (6.3) | 8 (14.5) | 18 (18.2) | | 6 (8.8) | | 8 (14.0) | | 5 (11.9) | | 8 (15.4) | | 5 (12.8) |
| **Number of prior lines of therapy^a^** | | | | | | | | | | | | | |
| 1 | - | - | - | | - | | 8 (14.0) | | 8 (19.0) | | 11 (21.2) | | 10 (25.6) |
| >1 | - | - | - | | - | | 49 (86.0) | | 34 (81.0) | | 41 (78.8) | | 29 (74.4) |
| **Refractory status** | | | | | | | | | | | | | |
| Refractory to IMiD agent | 12 (15.0) | 13 (23.6) | 66 (66.7) | | 45 (66.2) | | - | | - | | - | | - |
| Refractory to PI | 12 (15.0) | 12 (21.8) | 44 (44.4) | | 32 (47.1) | | - | | - | | - | | - |
| Refractory to Len | 8 (10.0) | 8 (14.5) | 49 (49.5) | | 34 (50.0) | | - | | - | | - | | - |
| Refractory to Bor | 11 (13.8) | 10 (18.2) | 41 (41.4) | | 29 (42.6) | | - | | - | | - | | - |
| Refractory to last regimen | 23 (28.8) | 26 (47.3) | 66 (66.7) | | 47 (69.1) | | - | | - | | - | | - |
| Refractory to Len at last regimen | 7 (8.8) | 8 (14.5) | 29 (29.3) | | 23 (33.8) | | - | | - | | - | | - |
| Refractory to Bor at last regimen | 9 (11.3) | 8 (14.5) | 23 (23.2) | | 15 (22.1) | | - | | - | | - | | - |
|  |  | |  | | |  | | | |  | | | |

Data are n (%) unless otherwise specified

^a^Per randomization by the IRT

^b^High-risk was defined as del(17p), or t(4;14), or t(14;16) by fluorescence in-situ hybridization. Cytogenetics was performed by a central laboratory with cut-offs of 50% for del(17p), 30% for t(4;14) and t(14;16)

Bor, bortezomib; CA, cytogenetic abnormality; d, dexamethasone; IMiD, immunomodulatory drug; IRT, Interactive Response Technology; Isa, isatuximab; ISS, International Staging System; K, carfilzomib; Len, lenalidomide; PI, proteasome inhibitor; R-ISS, Revised International Staging System

# Table S2. PFS probability by number of prior lines and refractory status

| **PFS probability (95% CI)^a^** | **Number of prior lines of therapy (IRT)** | | | | **Refractory status** | | | |
| --- | --- | --- | --- | --- | --- | --- | --- | --- |
|  | **1** | | **> 1** | | **Len-refractory** | | **Bor-refractory** | |
|  | **Isa-Kd (n = 80)** | **Kd (n = 55)** | **Isa-Kd (n = 99)** | **Kd (n = 68)** | **Isa-Kd (n = 57)** | **Kd (n = 42)** | **Isa-Kd (n = 52)** | **Kd (n = 39)** |
| 6 months | 0.94  (0.85–0.97) | 0.91  (0.79–0.96) | 0.89  (0.81–0.94) | 0.85  (0.73–0.91) | 0.85  (0.73–0.92) | 0.79  (0.63–0.89) | 0.88  (0.74–0.94) | 0.76  (0.59–0.87) |
| 12 months | 0.85  (0.75–0.92) | 0.73  (0.58–0.83) | 0.77  (0.66–0.84) | 0.67  (0.54–0.78) | 0.67  (0.52–0.78) | 0.60  (0.42–0.73) | 0.66  (0.50–0.78) | 0.60  (0.42–0.73) |
| 18 months | 0.77  (0.65–0.85) | 0.64  (0.49–0.76) | 0.68  (0.56– 0.76) | 0.45  (0.31–0.58) | 0.53  (0.38–0.66) | 0.31  (0.17– 0.47) | 0.63  (0.47–0.76) | 0.43  (0.26–0.59) |

^a^Estimated using the Kaplan-Meier method.

Bor, bortezomib; CI, confidence interval; d, dexamethasone; IRT, Interactive Response Technology; Isa, isatuximab; K, carfilzomib; Len, lenalidomide; PFS, progression-free survival

# Table S3. Safety summary by number of prior lines of therapy and refractory status

| **n (%)** | **Number of prior lines of therapy (IRT)** | | | | **Refractory status** | | | |
| --- | --- | --- | --- | --- | --- | --- | --- | --- |
|  | **1** | | **> 1** | | **Len-refractory** | | **Bor-refractory** | |
|  | **Isa-Kd (n = 79)** | **Kd (n = 54)** | **Isa-Kd (n = 98)** | **Kd (n = 68)** | **Isa-Kd (n = 57)** | **Kd (n = 42)** | **Isa-Kd (n =52)** | **Kd (n =39)** |
| Patients with any TEAE | 75 (94.9) | 51 (94.4) | 97 (99.0) | 66 (97.1) | 56 (98.2) | 40 (95.2) | 51 (98.1) | 36 (92.3) |
| Patients with any Grade ≥ 3 TEAE | 61 (77.2) | 35 (64.8) | 75 (76.5) | 47 (69.1) | 42 (73.7) | 26 (61.9) | 40 (76.9) | 26 (66.7) |
| Patients with any Grade 5 TEAE^a^ | 3 (3.8) | 0 | 3 (3.1) | 4 (5.9) | 2 (3.5) | 2 (4.8) | 1 (1.9) | 3 (7.7) |
| Patients with serious TEAE | 49 (62.0) | 26 (48.1) | 56 (57.1) | 44 (64.7) | 34 (59.6) | 21 (50.0) | 33 (63.5) | 24 (61.5) |
| Patients with any TEAE leading to definitive discontinuation | 7 (8.9) | 6 (11.1) | 8 (8.2) | 11 (16.2) | 4 (7.0) | 5 (11.9) | 2 (3.8) | 7 (17.9) |

^a^TEAE with fatal outcome during the treatment period

Bor, bortezomib; d, dexamethasone; IRT, Interactive Response Technology; Isa, isatuximab; K, carfilzomib; Len, lenalidomide; TEAE, treatment-emergent adverse event

# Table S4. TEAEs (occurring in ≥ 20% patients) by number of prior lines of therapy (safety population)

| **Number of prior lines of therapy (IRT)** | | **1 (N = 133)** | | | | | | | | | | | | **> 1 (N = 166)** | | | | | | | | | | | | | | | |
| --- | --- | --- | --- | --- | --- | --- | --- | --- | --- | --- | --- | --- | --- | --- | --- | --- | --- | --- | --- | --- | --- | --- | --- | --- | --- | --- | --- | --- | --- |
|  | | **Isa-Kd (n = 79)** | | | | | | **Kd (n = 54)** | | | | | | **Isa-Kd (n = 98)** | | | | | | | **Kd (n = 68)** | | | | | | | | |
| **TEAE, n (%)** | | **All grades** | | | **Grade ≥ 3** | | | **All grades** | | | **Grade ≥ 3** | | | **All grades** | | | | **Grade ≥ 3** | | | **All grades** | | | | **Grade ≥ 3** | | | | |
| Any class | | 75 (94.9) | | | 61 (77.2) | | | 51 (94.4) | | | 35 (64.8) | | | 97 (99.0) | | | | 75 (76.5) | | | 66 (97.1) | | | | 47 (69.1) | | | | |
| **Most common preferred terms in ≥ 20% of patients with isatuximab in any subgroup** | | | | | | | | | | | | | | | | | | | | | | | | | | | | | |
| Infusion-related reaction^a^ | 33 (41.7) | | | 1 (1.3) | | | 2 (3.7) | | | 0 | | | 48 (49.0) | | | 0 | | | | 2 (2.9) | | | | 0 | | | |  |  |
| Hypertension | 31 (39.2) | | | 17 (21.5) | | | 20 (37.0) | | | 11 (20.4) | | | 34 (34.7) | | | 19 (19.4) | | | | 18 (26.5) | | | | 13 (19.1) | | | |  |  |
| Upper respiratory tract infection | 31 (39.2) | | | 3 (3.8) | | | 18 (33.3) | | | 1 (1.9) | | | 33 (33.7) | | | 3 (3.1) | | | | 11 (16.2) | | | | 1 (1.5) | | | |  |  |
| Diarrhea | 27 (34.2) | | | 4 (5.1) | | | 16 (29.6) | | | 0 | | | 37 (37.8) | | | 1 (1.0) | | | | 19 (27.9) | | | | 3 (4.4) | | | |  |  |
| Fatigue | 22 (27.8) | | | 2 (2.5) | | | 11 (20.4) | | | 0 | | | 28 (28.6) | | | 4 (4.1) | | | | 12 (17.6) | | | | 1 (1.5) | | | |  |  |
| Insomnia | 21 (26.6) | | | 4 (5.1) | | | 12 (22.2) | | | 1 (1.9) | | | 21 (21.4) | | | 5 (5.1) | | | | 16 (23.5) | | | | 2 (2.9) | | | |  |  |
| Dyspnea | 21 (26.6) | | | 5 (6.3) | | | 11 (20.4) | | | 1 (1.9) | | | 28 (28.6) | | | 4 (4.1) | | | | 15 (22.1) | | | | 0 | | | |  |  |
| Pneumonia | 19 (24.1) | | | 13 (16.5) | | | 8 (14.8) | | | 6 (11.1) | | | 23 (23.5) | | | 16 (16.3) | | | | 16 (23.5) | | | | 9 (13.2) | | | |  |  |
| Bronchitis | 18 (22.8) | | | 3 (3.8) | | | 6 (11.1) | | | 1 (1.9) | | | 22 (22.4) | | | 1 (1.0) | | | | 9 (13.2) | | | | 0 | | | |  |  |
| Back pain | 16 (20.3) | | | 2 (2.5) | | | 14 (25.9) | | | 1 (1.9) | | | 23 (23.5) | | | 1 (1.0) | | | | 11 (16.2) | | | | 0 | | | |  |  |
| Cough | 14 (17.7) | | | 0 | | | 7 (13.0) | | | 0 | | | 21 (21.4) | | | 0 | | | | 10 (14.7) | | | | 0 | | | |  |  |
| Vomiting | 7 (8.9) | | | 1 (1.3) | | | 4 (7.4) | | | 1 (1.9) | | | 20 (20.4) | | | 1 (1.0) | | | | 7 (10.3) | | | | 0 | | | |  |  |
|  |  | | |  | | |  | | |  | | |  | | |  | | | |  | | | |  | | | |  |  |
| **Selected TEAEs** | | | | | | | | | | | | | | | | | | | | | | | | | | |  |  |  |
| Respiratory infection^b^ | 69 (87.3) | | | 27 (34.2) | | | 41 (75.9) | | | 13 (24.1) | | | 78 (79.6) | | | 30 (30.6) | | | | 49 (72.1) | | | | 16 (23.5) | | | |  |  |
| Thromboembolic events^c^ | 18 (22.8) | | | 5 (6.3) | | | 12 (22.2) | | | 4 (7.4) | | | 9 (9.2) | | | 2 (2.0) | | | | 8 (11.8) | | | | 3 (4.4) | | | |  |  |
| Cardiac failure^c^ | 8 (10.1) | | | 3 (3.8) | | | 1 (1.9) | | | 0 | | | 5 (5.1) | | | 4 (4.1) | | | | 7 (10.3) | | | | 5 (7.4) | | | |  |  |
| Ischemic heart disease^c^ | 5 (6.3) | | | 2 (2.5) | | | 0 | | | 0 | | | 3 (3.1) | | | 0 | | | | 5 (7.4) | | | | 2 (2.9) | | | |  |  |
| Second primary malignancy^b^ | 3 (3.8) | | | 1 (1.3) | | | 2 (3.7) | | | 2 (3.7) | | | 10 (10.2) | | | 3 (3.1) | | | | 4 (5.9) | | | | 2 (2.9) | | | |  |  |
|  |  | | |  | | |  | | |  | | |  | | |  | | | |  | | | |  | | | |  |  |
| **Hematological laboratory abnormalities** | **All grades** | | **Grade 3** | | | **Grade 4** | **All grades** | | **Grade 3** | | | **Grade 4** | | | **All grades** | | **Grade 3** | | **Grade 4** | | | **All grades** | **Grade 3** | | | **Grade 4** | | |  |
| Anemia | 78 (98.7) | | 13 (16.5) | | | 0 | 53 (98.1) | | 11 (20.4) | | | 0 | | | 98 (100) | | 26 (26.5) | | 0 | | | 68 (100) | 13 (19.1) | | | 0 | | |  |
| Neutropenia | 39 (49.4) | | 8 (10.1) | | | 0 | 22 (40.7) | | 5 (9.3) | | | 0 | | | 58 (59.2) | | 23 (23.5) | | 3 (3.1) | | | 31 (45.6) | 3 (4.4) | | | 1 (1.5) | | |  |
| Thrombocytopenia | 72 (91.1) | | 11 (13.9) | | | 3 (3.8) | 45 (83.3) | | 7 (13.0) | | | 1 (1.9) | | | 95 (96.9) | | 22 (22.4) | | 17 (17.3) | | | 62 (91.2) | 12 (17.6) | | | 9 (13.2) | | |  |

^a^Reported preferred term was infusion reaction in 34 patients and hypersensitivity in 1 patient in the 1 prior line subgroup; infusion reaction in 49 patients and cytokine release syndrome in 1 patient in the >1 prior line subgroup

^b^Groupings using customized MedDRA query: respiratory infection, second primary malignancy
^c^Groupings using standardized MedDRA query (narrow terms): thromboembolic events, cardiac failure, ischemic heart disease

d, dexamethasone; IRT, Interactive Response Technology; Isa, isatuximab; K, carfilzomib; MedDRA, Medical Dictionary for Regulatory Activities; TEAE, treatment-emergent adverse event

# Table S5. TEAEs (occurring in ≥ 20% patients) by refractory status (safety population)

| **Refractory status** | | | **Lenalidomide-refractory**  **(n = 99)** | | | | | | | | | | | **Bortezomib-refractory (n = 91)** | | | | | | | | | |  |
| --- | --- | --- | --- | --- | --- | --- | --- | --- | --- | --- | --- | --- | --- | --- | --- | --- | --- | --- | --- | --- | --- | --- | --- | --- |
|  | | | **Isa-Kd (n = 57)** | | | | | | **Kd (n = 42)** | | | | | **Isa-Kd (n = 52)** | | | | | **Kd (n = 39)** | | | | |  |
| **TEAE, n (%)** | | | **All grades** | | **Grade ≥ 3** | | | | **All grades** | | **Grade ≥ 3** | | | **All grades** | | **Grade ≥ 3** | | | **All grades** | | **Grade ≥ 3** | | |  |
| Any class | | | | 56 (98.2) | | 42 (73.7) | | | | 40 (95.2) | | 26 (61.9) | | | 51 (98.1) | | 40 (76.9) | | | 36 (92.3) | | 26 (66.7) | | |
|  | | | |  | |  | | | |  | |  | | |  | |  | | |  | |  | | |
| **Most common preferred terms in ≥ 20% of patients with isatuximab in any subgroup** | | | | | | | | | | | | | | | | | | | | | | | | |
| Infusion-related reaction^a^ | | | 29 (50.9) | | 0 | | | | 2 (4.8) | | 0 | | | 25 (48.1) | | 0 | | | 2 (5.1) | | 0 | | |  |
| Diarrhea | | | 23 (40.4) | | 2 (3.5) | | | | 9 (21.4) | | 0 | | | 16 (30.8) | | 1 (1.9) | | | 11 (28.2) | | 1 (2.6) | | |  |
| Cough | | | 19 (33.3) | | 0 | | | | 6 (14.3) | | 0 | | | 10 (19.2) | | 0 | | | 3 (7.7) | | 0 | | |  |
| Hypertension | | | 19 (33.3) | | 11 (19.3) | | | | 6 (14.3) | | 5 (11.9) | | | 17 (32.7) | | 7 (13.5) | | | 12 (30.8) | | 8 (20.5) | | |  |
| Fatigue | | | 18 (31.6) | | 3 (5.3) | | | | 4 (9.5) | | 0 | | | 12 (23.1) | | 1 (1.9) | | | 5 (12.8) | | 1 (2.6) | | |  |
| Back pain | | | 17 (29.8) | | 1 (1.8) | | | | 10 (23.8) | | 0 | | | 14 (26.9) | | 1 (1.9) | | | 7 (17.9) | | 0 | | |  |
| Dyspnea | | | 17 (29.8) | | 2 (3.5) | | | | 7 (16.7) | | 1 (2.4) | | | 11 (21.2) | | 4 (7.7) | | | 6 (15.4) | | 0 | | |  |
| Insomnia | | | 17 (29.8) | | 4 (7.0) | | | | 9 (21.4) | | 2 (4.8) | | | 9 (17.3) | | 2 (3.8) | | | 5 (12.8) | | 2 (5.1) | | |  |
| Upper respiratory tract infection | | | 17 (29.8) | | 1 (1.8) | | | | 5 (11.9) | | 1 (2.4) | | | 16 (30.8) | | 2 (3.8) | | | 11 (28.2) | | 1 (2.6) | | |  |
| Constipation | | | 16 (28.1) | | 1 (1.8) | | | | 5 (11.9) | | 0 | | | 6 (11.5) | | 0 | | | 3 (7.7) | | 0 | | |  |
| Bronchitis | | | 15 (26.3) | | 0 | | | | 6 (14.3) | | 0 | | | 12 (23.1) | | 1 (1.9) | | | 4 (10.3) | | 0 | | |  |
| Pneumonia | | | 13 (22.8) | | 10 (17.5) | | | | 7 (16.7) | | 4 (9.5) | | | 10 (19.2) | | 7 (13.5) | | | 11 (28.2) | | 7 (17.9) | | |  |
| Arthralgia | | | 12 (21.1) | | 0 | | | | 3 (7.1) | | 1 (2.4) | | | 6 (11.5) | | 0 | | | 2 (5.1) | | 0 | | |  |
| Asthenia | | | 12 (21.1) | | 0 | | | | 7 (16.7) | | 1 (2.4) | | | 7 (13.5) | | 0 | | | 5 (12.8) | | 1 (2.6) | | |  |
| Nausea | | | 12 (21.1) | | 0 | | | | 2 (4.8) | | 0 | | | 8 (15.4) | | 0 | | | 5 (!2.8) | | 0 | | |  |
| Peripheral sensory neuropathy | | | 12 (21.1) | | 0 | | | | 6 (14.3) | | 0 | | | 5 (9.6) | | 0 | | | 3 (7.7) | | 0 | | |  |
|  | | |  | |  | | | |  | |  | | |  | |  | | |  | |  | | |  |
| **Selected TEAEs** | |  | | | | |  | | | | | | | | | | | | | | | | |  |
| Respiratory infection^b^ | | | 43 (75.4) | | 17 (29.8) | | | | 28 (66.7) | | 7 (16.7) | | | 39 (75.0) | | 19 (36.5) | | | 27 (69.2) | | 10 (25.6) | | |  |
| Thromboembolic events^c^ | | | 8 (14.0) | | 2 (3.5) | | | | 4 (9.5) | | 2 (4.8) | | | 7 (13.5) | | 0 | | | 7 (17.9) | | 3 (7.7) | | |  |
| Cardiac failure^c^ | | | 3 (5.3) | | 2 (3.5) | | | | 3 (7.1) | | 1 (2.4) | | | 3 (5.8) | | 3 (5.8) | | | 4 (10.3) | | 3 (7.7) | | |  |
| Ischemic heart disease^c^ | | | 3 (5.3) | | 1 (1.8) | | | | 1 (2.4) | | 0 | | | 1 (1.9) | | 0 | | | 1 (2.6) | | 1 (2.6) | | |  |
| Second primary malignancy^b^ | | | 5 (8.8) | | 1 (1.8) | | | | 3 (7.1) | | 2 (4.8) | | | 6 (11.5) | | 1 (1.9) | | | 2 (5.1) | | 1 (2.6) | | |  |
|  | | |  | |  | | | |  | |  | | |  | |  | | |  | |  | | |  |
| **Hematological laboratory abnormalities** | | | **All grades** | | **Grade 3** | | | **Grade 4** | **All grades** | | **Grade 3** | | **Grade 4** | **All grades** | | **Grade 3** | | **Grade 4** | **All grades** | | **Grade 3** | | **Grade 4** |  |
| Neutropenia | | | 36 (63.2) | | 17 (29.8) | | | 1 (1.8) | 23 (54.8) | | 4 (9.5) | | 1 (2.4) | 30 (57.7) | | 13 (25.0) | | 1 (1.9) | 20 (51.3) | | 4 (10.3) | | 1 (2.6) |  |
| Anemia | | | 57 (100) | | 14 (24.6) | | | 0 | 41 (97.6) | | 8 (19.0) | | 0 | 52 (100) | | 14 (26.9) | | 0 | 39 (100) | | 13 (33.3) | | 0 |  |
| Thrombocytopenia | | | 55 (96.5) | | 15 (26.3) | | | 11 (19.3) | 37 (88.1) | | 7 (16.7) | | 7 (16.7) | 49 (94.2) | | 10 (19.2) | | 7 (13.5) | 32 (82.1) | | 5 (12.8) | | 4 (10.3) |  |
|  | | |  | |  | | |  |  | |  | |  |  | |  | |  |  | |  | |  |  |

^a^Reported preferred term was infusion reaction in 30 patients and cytokine release syndrome in 1 patient in the lenalidomide-refractory subgroup; infusion reaction in 27 patients in the bortezomib-refractory subgroup

^b^Groupings using customized MedDRA query: respiratory infection, second primary malignancy

^c^Groupings using standardized MedDRA query (narrow terms): thromboembolic events, cardiac failure, ischemic heart disease

d, dexamethasone; Isa, isatuximab; K, carfilzomib; MedDRA, Medical Dictionary for Regulatory Activities; TEAE, treatment-emergent adverse event

# References

1. Moreau P, Dimopoulos M, Mikhael J, et al. Isatuximab, carfilzomib, and dexamethasone in relapsed multiple myeloma (IKEMA): a multicentre, open-label, randomised phase 3 trial *Lancet.* 2021;397(10292):2361-2371.

2. Kumar S, Paiva B, Anderson KC, et al. International Myeloma Working Group consensus criteria for response and minimal residual disease assessment in multiple myeloma. *Lancet Oncol.* 2016;17(8):e328-e346.
